# Characterization of the microbiome of nipple aspirate fluid of breast cancer survivors

**DOI:** 10.1038/srep28061

**Published:** 2016-06-21

**Authors:** Alfred A. Chan, Mina Bashir, Magali N. Rivas, Karen Duvall, Peter A. Sieling, Thomas R. Pieber, Parag A. Vaishampayan, Susan M. Love, Delphine J. Lee

**Affiliations:** 1 Dirks/Dougherty Laboratory for Cancer Research, Department of Translational Immunology, John Wayne Cancer Institute at Providence Saint John’s Health Center, Santa Monica, CA, USA; 2 Biotechnology and Planetary Protection Group, Jet Propulsion Laboratory, California Institute of Technology, Pasadena, CA, USA; 3 Division of Endocrinology and Metabolism, Medical University of Graz, Graz, Austria; 4 Breast Center at the University of California Los Angeles (UCLA), Westwood, Los Angeles, CA, USA; 5 Dr. Susan Love Research Foundation, Encino, CA, USA

## Abstract

The microbiome impacts human health and disease. Until recently, human breast tissue and milk were presumed to be sterile. Here, we investigated the presence of microbes in the nipple aspirate fluid (NAF) and their potential association with breast cancer. We compared the NAF microbiome between women with a history of breast cancer (BC) and healthy control women (HC) using 16S rRNA gene amplicon sequencing. The NAF microbiome from BC and HC showed significant differences in community composition. Two Operational Taxonomic Units (OTUs) showed differences in relative abundances between NAF collected from BC and HC. In NAF collected from BC, there was relatively higher incidence of the genus *Alistipes.* By contrast, an unclassified genus from the *Sphingomonadaceae* family was relatively more abundant in NAF from HC. These findings reflect the ductal source DNA since there were no differences between areolar skin samples collected from BC and HC. Furthermore, the microbes associated with BC share an enzymatic activity, Beta-Glucuronidase, which may promote breast cancer. This is the first report of bacterial DNA in human breast ductal fluid and the differences between NAF from HC and BC. Further investigation of the ductal microbiome and its potential role in breast cancer are warranted.

The human microbiome is the term applied to the universe of microbes that inhabit our skin and mucosal surfaces. Epidemiologic studies suggest that the human microflora contributes to 16% or more of worldwide malignancies^1^,^2^,^3^. Increased cancer risk is associated with the presence of chronic, persistent, and dysregulated inflammation^1^,^3^,^4^. Many of the studies on microbes in relation to cancer have focused on the gut microflora. For instance, infection with *Helicobacter pylori,* a gram-negative bacterium that selectively colonizes the gastric epithelium and induces gastric inflammation, is correlated with a higher incidence of gastric cancer^5^. Further, emerging evidence suggests that infection with *Fusobacterium nucleatum,* a common member of the oropharyngeal flora and a pathogenic agent involved in gingival and periodontal disease, is associated with the development of human colorectal cancer^6^,^7^. Bacteria present near the tumor site are part of the tumor microenvironment. On one hand, the microbiome might promote malignancy by inducing chronic inflammation, by altering the balance of host cell proliferation and death, and by triggering uncontrolled innate and adaptive immune responses^8^. On the other hand, certain microbes might play a preventative role in breast carcinogenesis by affecting levels of estrogen or by promoting antitumor immunity and immune surveillance^9^.

The National Institutes of Health (NIH) Human Microbiome Project (HMP) has a reference collection of bacterial genomes associated with multiple body sites from healthy human adults. Although an important number of anatomical sites were sampled for their microbiome, the human breast was omitted^10^, presumably because most would claim that, at steady state, the breast tissue is sterile and devoid of any bacterial presence. However, the presence of six to eight ductal openings at the surface of the human nipple allows microbes from the environment, skin, and mouth (through sexual activity and breast feeding) to access the breast ductal system^11^. In fact, microbes present in human breast milk and breast tissue have recently been characterized using next-generation sequencing and pan-pathogen array technologies^9^,^12^,^13^,^14^,^15^.

Until now, the potential role of the local breast ductal microbiome with breast cancer has not been explored. In this study, we used 16S rRNA gene sequencing to characterize the microorganisms present in nipple aspirate fluid (NAF). NAF is constantly secreted and absorbed by the epithelial cells lining the breast ducts and can be obtained non-invasively from at least one duct in a majority of women by applying negative pressure with a syringe attached to a suction cup^16^. NAF collected from breast cancer patients has been shown to have a significantly different proteomic profile compared to NAF collected from healthy volunteers^17^. Here, we collected NAF from healthy control women (HC) and women with a history of breast cancer (BC) (all were ductal carcinomas) to investigate the breast ductal microbiome.

## Results

### Nipple skin microbiome from HC vs. BC

**Figure 1:**
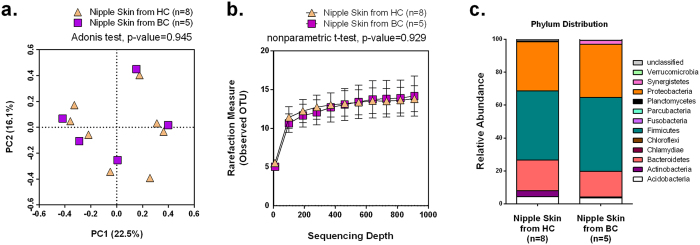
Nipple skin microbial composition. (**a**) PCoA plot using Bray-Curtis dissimilarity based on genus-level OTUs from nipple skin swab samples collected from either HC or BC. (**b**) Number of bacterial OTUs observed on the nipple skin swabs as a function of sequencing depth as assessed by diversity rarefaction curves. The difference in diversity was compared by a non-parametric t-test using the average of observed OTUs randomly sampled ten times at 918 sequencing depth. Error bars represent standard deviation. (*c*) Bar chart depicts the average phylum-level percentages of the nipple skin microbiome measured from HC and BC.

Nipple/areola skin was sampled with a sterile cotton swab as a control to compare to NAF. DNA extracted from the nipple skin samples was sequenced, the reads were clustered into unique Operation Taxonomic Units (OTUs), and then the OTUs classified to the genus taxonomic level. The relative differences amongst the nipple skin communities were calculated using the Bray-Curtis index and graphically visualized using Principal Coordinates Analysis (PCoA), whereby a shorter distance between points indicates increasing similarity in microbial composition. Though the skin varies according to body site^18^ and is expected to randomly vary across individuals, we hypothesized that the nipple skin microbiome would be independent of breast cancer history. The nipple skin microbiome from HC (n = 8) and BC (n = 5) did not cluster separately and did not have a significant difference in bacterial composition (Adonis, p-value = 0.945) (Fig. 1a). The rarefaction curves, which plot the number of unique species as a function of the number of reads sampled, reached a plateau for the nipple skin samples as well as for the other sample types (post-Betadine skin and NAF), indicating that our sampling depth provided sufficient coverage to capture most members of the bacterial communities (Figs 1b). The bacterial diversity was not significantly different between the nipple skin sampled from HC vs. BC (nonparametric t-test, p-value = 0.929, Fig. 1b).

In nipple skin samples from both HC and BC, bacterial composition at the phyla level was predominantly Proteobacteria (average 36.5%), Firmicutes (average 33.8%), and Bacteroidetes (average 19.5%) (Fig. 1c). Of all the OTUs in the nipple skin samples, *Alistipes* (recently reclassified *Bacteroides putredinis*^19^) was the most abundant (average 11.8%), followed by an unclassified genus from the *Sphingomonadaceae* family (average 11.3%), *Rhizobium* (average 6.7%), and an unclassified family from Acidobacteria Gp4 (average 4.9%). None of the OTUs from the skin swabs were significantly different when comparing their relative abundance between the HC and BC groups (Kruskal-Wallis test, Supplemental Table S1). In summary, the nipple skin microbiome from HC and BC were not significantly distinguishable by their community composition, their diversity, or their individual OTUs, indicating that the nipple skin microbiome is independent of breast cancer history (Fig. 1).

### Microbiome in post-Betadine treated skin swab from HC vs. BC

**Figure 2:**
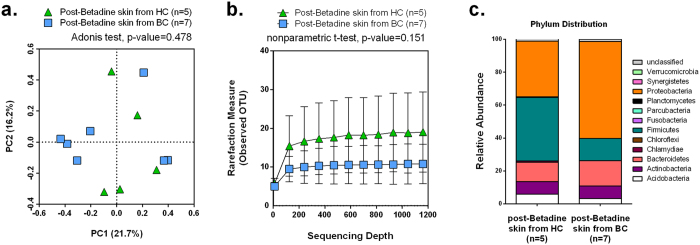
Post-Betadine skin microbial composition. (**a**) PCoA plot using Bray-Curtis dissimilarity based on genus-level OTUs from post-Betadine skin samples from either HC (n = 5) or BC (n = 7). (**b**) Number of bacterial OTUs observed on the post-Betadine skin as a function of sequencing depth as assessed by diversity rarefaction curves. The difference in diversity was compared by a non-parametric t-test using the average of observed OTUs randomly sampled ten times at 1164 sequencing depth. Error bars represent standard deviation. (**c**). Bar chart depicts the average phylum-level percentages of the post-Betadine skin swab microbiome measured from HC and BC.

Betadine is an iodine-based broad spectrum disinfection agent used to clean skin before surgeries and other procedures. The nipple skin was treated with Betadine to avoid contamination of NAF samples with normal nipple skin flora. We found that Betadine leaves a small residual flora that is detected by the extremely sensitive methodology used in this study. As a baseline control, we collected post-Betadine samples of the nipple skin to characterize the residual microflora. Reads from the post-Betadine skin swabs were clustered into OTUs and classified to the genera level. We calculated the Bray-Curtis dissimilarity index and performed PCoA to visualize community-wide differences amongst the post-Betadine skin swabs. The post-Betadine skin microbiome from HC (n = 5) and BC (n = 7) did not separate into distinct clusters (Adonis, p-value = 0.478) (Fig. 2a), and the bacterial diversity between the two groups were not significantly different (nonparametric t-test, p-value = 0.151) (Fig. 2b). The post-Betadine swab microbiome is primarily composed of bacteria belonging to the phylum Proteobacteria (average 49.4%), Firmicutes (average 23.8%), and Bacteroidetes (average 13.0%) (Fig. 2c). None of the OTUs was significantly different between the post-Betadine skin samples collected from HC and BC (Kruskal-Wallis test, Supplemental Table S2). As expected, the post-Betadine nipple skin microbiome from HC vs. BC was not significantly different by comparing community composition, by comparing species diversity, or by comparing relative OTU abundances, indicating that the post-Betadine controls are independent of breast cancer history (Fig. 2).

### NAF microbial composition from HC vs. BC

**Figure 3:**
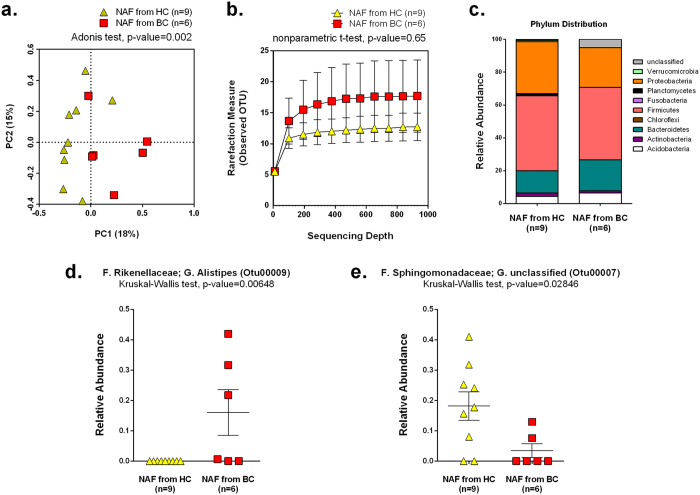
NAF microbial composition. (**a**) PCoA plot of Bray-Curtis dissimilarity based on genus-level OTUs of NAF collected from either HC (n = 9) or BC (n = 6). (**b**) Number of bacterial OTUs observed in the NAF as a function of sequencing depth as assessed by diversity rarefaction curves. The difference in diversity was compared by a non-parametric t-test using the average of observed OTUs randomly sampled ten times at 934 sequencing depth. Error bars represent standard deviation. (**c**) Bar chart depicts the average phylum-level percentages of the NAF microbiome measured from HC and BC. (**d**) Strip-plot comparing relative abundances of the genus *Alistipes* (Otu00009) between HC and BC group. (**e**) Strip-plot comparing relative abundances of the unclassified genus from the *Sphingomonadaceae* family (Otu00007) between HC and BC group.

To assess whether there is a difference in microbial composition between the NAF from HC and BC, we calculated sample-to-sample variations by Bray-Curtis dissimilarity and then visualized the compositional differences by PCoA (Fig. 3a). We found that the NAF microbiome from HC and BC clustered separately with a significant difference (Adonis, p-value = 0.002). The Adonis test indicates that having had a history of breast cancer significantly affects the NAF microbial composition and explains approximately 13.5% of the variation among the samples. The bacterial diversity of NAF was not significantly different between those collected from HC vs. those from BC (nonparametric t-test, p-value = 0.65) (Fig. 3b).

The most abundant bacteria in NAF samples were those belonging to the phylum Firmicutes (averaging 42.1%), Proteobacteria (averaging 32.9%), and Bacteroidetes (averaging 14.5%) (Fig. 3c). Two OTUs showed differences in relative abundance between NAF from HC and from BC by Kruskal-Wallis test (Supplemental Table S3). The genus *Alistipes* (Otu00009) was only present in the NAF from BC (p-value = 0.0068) (Fig. 3d). In contrast, an unclassified genus from the family Sphingomonadaceae (Otu00007) was present in NAF from both HC and BC, but was relatively more abundant in the NAF from HC compared to BC (p-value = 0.02846) (Fig. 3e). Unlike the microbiome from the nipple skin and the post-Betadine controls, the microbiome from NAF demonstrated significant clustering between HC and BC with two differentially abundant OTUs between the two health states.

### Microbial composition from nipple skin vs. NAF

**Figure 4:**
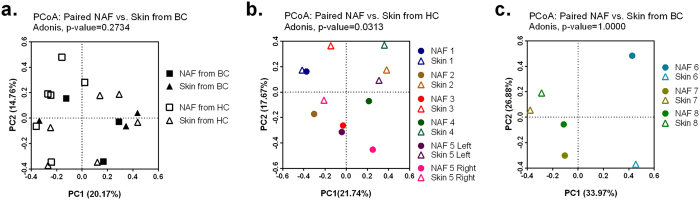
Nipple skin and NAF microbial composition comparison. (**a**) PCoA plot of Bray-Curtis dissimilarity between paired skin and NAF samples collected from both HC and BC (n = 9). (**b**) PCoA plot of Bray-Curtis dissimilarity between paired skin and NAF samples collected from HC (n = 6). (**c**) PCoA plot of Bray-Curtis dissimilarity between paired skin and NAF samples collected from BC (n = 3). All subjects with sequences obtained from both skin and NAF are shown. The paired community wide testing was performed using Adonis test with the strata parameter to allow permutation only within patients and not across.

To address whether the microbes in NAF correspond to those on the overlying nipple and areolar skin, we compared the microbiome between the nipple skin and NAF. Because of the variability of microbiome across individuals^10^, the analysis was limited to paired NAF and corresponding nipple skin samples collected from the same breast. We calculated the sample-to-sample variation by the Bray-Curtis dissimilarity and visualized it using PCoA (Fig. 4a). There was no compositional difference between the microbes present on nipple skin and in NAF when combining all HC and BC samples (Adonis, p-value = 0.2734) (Fig. 4a). However, a history of breast cancer is a confounding variable, so the samples from HC group and BC group were analyzed separately and the Bray-Curtis dissimilarity recalculated for each group (Fig. 4b,c).

Within the HC sample pairs (n = 6), the nipple skin and NAF microbiome were significantly different by paired Adonis test using the strata parameter (p-value = 0.0313). However, there was no difference in bacterial diversity (paired t-test, p-value = 0.62), and none of the OTUs were significantly different between the nipple skin and NAF groups (Paired Wilcoxon signed-rank test, Supplemental Table S4).

Within the BC samples (n = 3), the paired comparison between the nipple skin and NAF microbiome was not significantly different (Adonis, p-value = 1.000). Comparing the nipple skin and NAF sample types from BC, there was no difference in bacterial diversity (paired t-test, p-value = 0.52), and none of the OTUs were significantly different (Paired Wilcoxon signed-rank test, Supplemental Table S5).

### Functional prediction based on NAF microbiome comparing HC vs. BC

**Figure 5:**
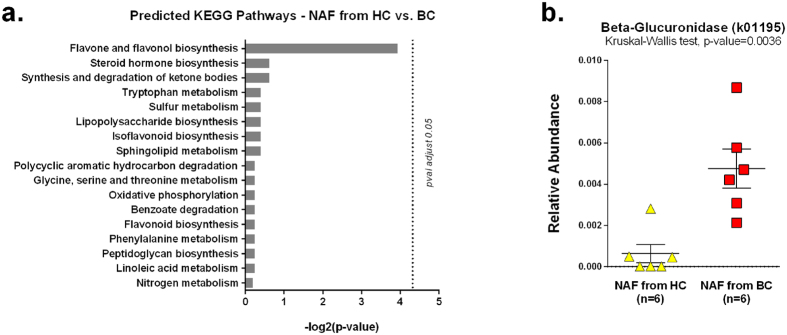
PICRUSt-predicted metagenomes between NAF from HC vs. BC. (**a**) Negative log2 of adjusted-pvalues from Kruskal-Wallis test comparing the relative abundances of 18 selected KEGG pathways in NAF from HC (n = 6) vs. BC (n = 6). The NAF microbiome was normalized by copy numbers before the PICRUSt metagenomic inference. Relative abundances were normalized to a threshold of 26359 counts (lowest of the samples) before the Kruskal-Wallis test. (**b**) Strip-plot comparing the relative abundance of the KEGG gene “Beta-Glucuronidase” between NAF from HC vs. BC. Beta-Glucuronidase is the only predicted KEGG ortholog that contributes to the “Flavone and Flavonol Biosynthesis” pathway. Beta-Glucuronidase is predicted by a composite of 8 OTUs shown in Table 2.

To investigate whether the microbes present in the breast ducts have a metabolic activity related to malignancy or health, we examined the gene sequences associated with 18 selected KEGG metabolic pathways that have been implicated in colon cancer^20^,^21^,^22^,^23^,^24^,^25^,^26^,^27^ using the PICRUSt package^28^. None of the 18 selected KEGG pathways were statistically significant at 5% alpha level after correcting for multiple hypothesis testing. The pathway “flavone and flavonol biosynthesis (ko00944)” produced the lowest p-value (Kruskal-Wallis test, adjusted p-value = 0.066) (Fig. 5a). Of the 12 KEGG orthologs that comprise the “flavone and flavonol biosynthesis” pathway, “Beta-Glucuronidase (k01195)” was the only KEGG bacterial ortholog predicted by the NAF microbiome. Furthermore, the gene “Beta-Glucuronidase” is inferred from a combination of eight OTUs (Table 1) and the combined relative abundances of these eight OTUs were statistically higher in NAF from BC than HC (Kruskal-Wallis test, p-value = 0.0036) (Fig. 5b).

## Discussion

This study establishes the existence of a microbiome in the breast ductal system, by detecting the presence of bacterial DNA in NAF. Analysis of the beta-diversity demonstrates that the NAF microbial community composition is different in women having had a history of breast cancer. Previous microbiome studies of breast tissue have described bacteria belonging mainly to the phylum Proteobacteria, Firmicutes, Actinobacteria, and Bacteroidetes^9^,^12^,^15^. Our studies on NAF show a similar microbial composition, with Firmicutes, Proteobacteria, and Bacteroidetes. While previous studies on NAF have attempted to define biomarkers to help early detection and prevention of breast cancer using cellular morphology, protein expression, growth factors, and hormones in the intraductal microenvironment^29^, this is the first study to identify bacteria in the ductal system.

While microbiome studies of NAF have never been reported, breast milk has been studied for its community composition^13^,^14^,^30^. Both NAF and milk are secreted by the epithelial cells lining the breast ducts. Breast milk contains bacteria that are orally transmitted to the breastfed infant with a key function to populate and establish the neonate’s gastrointestinal flora^14^. The bacteria present in milk belong mostly to the phylum Firmicutes (*Staphylococcus, Clostridium, Lactobacillus*), Actinobacteria (*Propionibacterium, Corynebacterium*), Proteobacteria (*Ralstonia, Sphingomonas, Pseudomonas, Bradyrhizobiaccea*), and Bacteroidetes (*Prevotella*)^13^,^14^,^30^. Similarly, in NAF samples from both HC and BC, we found *Clostridium* and *Prevotella*, as well as an unclassified genus from the *Sphingomonadaceae* family. Thus, the NAF and milk microbiomes exhibit some overlap.

Though the existence of bacteria in breast tissue has previously been described, the current study establishes the presence of bacterial DNA in the breast ductal system and thereby raises the question ‘where do breast ductal bacteria originate?’^9^,^12^. Ductal spaces may be populated by bacteria from oral sources, resulting from sexual activity or breast-feeding. An entero-mammary pathway by which some bacteria from the gut could reach the mammary gland via an endogenous route has been postulated by others^31^. Alternatively, it is tempting to speculate that skin bacteria from the nipple surface might reach the breast ducts through the ductal orifices via retrograde migration since the nipple skin and NAF microbiome had no significant OTUs by paired Wilcoxon signed-rank test. However, amongst the samples from HC, the nipple skin and the NAF had significantly different community composition. Additional studies with larger sample sizes are required to investigate this preliminary trend.

Two individual OTUs showed differences in relative abundances between NAF from HC and BC. First, the genus *Alistipes* (Otu00009) was present only in the NAF from BC, and was absent in the NAF collected from HC. Alistipes has been included among other bacteria to have an increased relative abundance in colorectal cancer^32^. Another organism of interest, an unclassified genus from the *Sphingomonadaceae* family (Otu00007), was relatively more abundant in NAF from HC than from BC. The consensus sequence from this OTU was queried against the NCBI “refseq_rna” using the default BLAST algorithm (Basic Local Alignment Search Tool)^33^. The OTU matched two strains of *Sphingobium yanoikuyae* (NR_113730.1 and NR_11524.1) with 100% identity. Notably, in our previous study, four out of the eight OTUs enriched in paired normal compared to estrogen-receptor positive (ER+) breast tumor tissue belonged to the *Sphingomonadaceae* family with *Sphingobium yanoikuyae* having the greatest statistical significance^9^.

Members of the *Sphingomonadaceae* family are known for their ability to degrade aromatic hydrocarbons^34^. Polycyclic aromatic hydrocarbons have been associated with breast cancer^35^. It is interesting that a microbe capable of degrading aromatic hydrocarbons, such as estrogen, would be comparatively higher in the healthy state and lower in the estrogen-dependent breast tumor^36^. This may be especially relevant because the sex hormones have far higher concentration in the nipple aspirate fluid than in the serum^16^. In the present study, the metabolism of aromatic hydrocarbons by *Sphingomonadaceae* may have a protective role and account for the greater abundance of these organisms in the NAF from healthy women, a possibility that warrants further investigation.

If bacterial metabolism were related to breast cancer, then certain metabolic pathways would be discernable between the NAF collected from HC and BC. PICRUSt analysis infers the microbial gene contents and their functional profiles using a database of annotated sequences. None of the 18 selected KEGG pathways were statistically significant at 5% alpha level after correcting for multiple hypothesis testing. The pathway “flavone and flavonol biosynthesis (ko00944)” produced the lowest p-value (Kruskal-Wallis test, adjusted p-value = 0.066) (Fig. 5a). Of the 12 KEGG orthologs that comprise the “flavone and flavonol biosynthesis” pathway, “Beta-Glucuronidase (k01195)” was the only KEGG bacterial ortholog predicted by the NAF microbiome. The predicted Beta-Glucuronidase levels were higher in NAF from BC than from HC.

Beta-Glucuronidase is known to be a procarcinogenic enzyme in gastrointestinal cancer, and has been used as a marker to evaluate dietary effects on colon carcinogenesis^37^,^38^. In addition, bacterial Beta-Glucuronidase activity has also been reported to be higher in patients with colon cancer than healthy controls^39^. Notably, the NAF OTUs predictive of Beta-Glucuronidase levels (Table 1) were different from those reported in colon cancer, which are *Enterobacteriaceae* family members and some Firmicutes genera^40^. This preliminary finding hints at the potential role of bacterial Beta-Glucuronidase in relation to breast cancer. Finally, this analysis suggests that defining the microbiome by functional gene analysis may be more physiologically relevant than simply measuring individual OTU differences based on taxonomic affiliations.

Implication of Beta-Glucuronidase in breast cancer is not a new idea. One way estrogen is excreted is by conjugating it with glucuronic acid, making it water soluble for excretion^41^. When glucuronidate-conjugated estrogen reaches a site rich with Beta-Glucuronidase from microorganisms or from inflammation (human lysosomes contain Beta-Glucuronidase), the enzyme reverses the conjugation and leaves biologically active estrogen to accumulate at the site^42^. In a rat model for breast cancer, inhibition of Beta-Glucuronidase with calcium D-glucarate reduces breast cancer incidence by lowering endogenous levels of estradiol^43^. Some have even postulated the use of calcium D-glucarate supplementation to reduce the cancer risk of some individuals^44^. There are no previous reports of elevated Beta-Glucuronidase in breast ductal fluid. Our preliminary finding provokes an intriguing hypothesis that bacterial Beta-Glucuronidase may deconjugate glucuronidate-conjugated estrogen present in breast tissue.

Apart from metabolic activity, microbes may contribute to the multifactorial susceptibility to breast cancer^2^. The epithelial cells that line the breast ducts act as a barrier to the outside world and, unless breached, separate the NAF microbiome from that of the tissue. In addition, the ductal epithelium has the capability to sense microbial-derived signals through Toll-Like Receptors (TLRs) and NOD-like receptors (NLRs) expressed at their surfaces^45^. While previous studies have demonstrated that the activation of the TLR2-MyD88 pathway in mammary and gastrointestinal epithelial cells leads to tumor growth and promotes carcinogenesis^45^,^46^, TLR5 activation with flagellin inhibits cancer cell growth and mediates potent anti-tumor activity in breast cancer^36^. Interestingly, *Sphingobium yanoikuyae* is motile with a single polar flagellum^47^, providing a ligand for the TLR5 pathway. Further work is warranted to investigate the potential interaction of the breast ductal microbiome and the immune response as it relates to breast cancer.

This study was limited to the microbiome obtained from ducts that produced NAF, while ducts that did not produce NAF were not studied. In any given individual, NAF was produced by one or more ducts, and NAF from multiple ducts can vary within an individual^48^. Future investigations should focus on individual ducts from normal subjects and subjects with limited early breast disease such as ductal carcinoma *in situ* (DCIS). This would provide valuable insight into microbiome variations between malignant and healthy ducts and their relation to breast cancer.

In conclusion, this study demonstrates the presence of microbes in NAF, shows that the microbiome of NAF from HC and BC are significantly different, identifies particular organisms that are differentially present in HC and BC, and provides a metabolic insight into possible mechanisms for the association between breast microflora and malignancy. Since the treatment information for all BC was not available, we cannot rule out the possibility that another factor BC have in common, such as radiation or other therapy for breast cancer could have contributed to our finding. Further studies should explore how bacteria are associated with breast cancer.

## Methods

### Study population

All experiments involving the use of human tissue samples were performed in accordance with the Common Rule (45 CFR 46), ICH E6 GCP guidance as well as the Western IRB’s requirements for consenting subjects. Written informed consent was obtained from all human subjects. All experimental protocols were approved by the Western Institutional Review Board (protocol number 20111656) and specimens received in the Lee laboratory from Dr. Susan Love Research Foundation were de-identified and accepted under an IRB exemption approved by John Wayne Cancer Institute Regulatory affairs.

48 women, 23 healthy control women (HC) and 25 with a history of breast cancer (BC), 18 years or older with at least one intact nipple, provided informed consent and were recruited in the Love Army of Women under a protocol approved by the Western Institutional Review Board. All of the breast cancers were ductal carcinomas. Subjects were excluded if they had been diagnosed with metastatic breast cancer; taken antibiotic therapy less than six months from the date of consent; taken oral contraceptives, hormone replacement therapy, any form of estrogen, any selective estrogen receptor modulators, or any aromatase inhibitors within 12 months from the date of consent; were currently lactating or had lactated within 12 months from the date of consent; had any known abnormal levels of sex hormones or prolactin; had received chemotherapy or radiation less than 12 months from the date of consent; had any subareolar or other surgery (papilloma resections, biopsies, or fine needle aspirations) within two centimeters of the nipple; had any active infections or inflammation in the breast; or were unwilling to sign an informed consent.

### Sample collection

Prior to NAF collection, subjects warmed their breasts with a heating pad, placed outside the hospital gown, for approximately 20 minutes and then massaged their breasts for approximately five minutes. The pad does not come into contact with the subject’s skin. Skin sampling was performed according to the methods of Grice *et al*., as described in their paper and after communication with the authors. First, we collected a swab of the nipple skin by rubbing a sterile cotton swab (Thermo Fisher Scientific, Lexena, KS) over the surface of the nipple and then the areola once in an expanding circular motion. Next, the nipple was de-keratinized using a mild abrasive gel (Nuprep, D.O. Weaver & Co., Aurora, CO) followed by the application of Betadine® solution (Purdue Products, Wilson, NC) to sterilize the skin surface. After the preparation with Betadine, a sterile cotton swab was used to collect a post-Betadine nipple skin sample. The entire procedure was performed with open surgery level sterility with the surgeon wearing a scrub suit with cap, gown, shoe covers, mask, and gloves. To elicit NAF, a suction cup fitted with a 20 ml syringe was used to create negative pressure. NAF was collected with a sterile cotton swab. For HC, collection of NAF was attempted on both breasts, but for BC, only the contralateral breast was sampled since the ductal system for the breast that had previously been treated for breast cancer would be interrupted. Sampling was performed by the same clinical research team at one location and all the samples in the analysis were collected by one physician. The protocol for NAF collection has been included in Supplemental Method.

### Preparation of samples for 16S rRNA sequencing

Cotton swab samples of the nipple skin and NAF were immediately placed in sterile RNase/DNase free Eppendorf tubes and kept at −80 °C until genomic DNA (gDNA) extraction. gDNA extraction was performed with a QIAamp DNA Mini Kit (Qiagen) according to the manufacturer’s instruction. Samples were extracted in batches, with random selection to avoid any batch effects. We used empty Eppendorf tube controls running along all preparations to evaluate potential contamination. Isolated gDNA was submitted to Second Genome Inc. for 16S-V4 rRNA gene amplicon sequencing. DNA from each sample was amplified using Caporaso primers tailed with sequences to incorporate flow cell adapters and indexing barcodes. The sequence for the forward primer (F515) was 5′AATGATACGGCGACCACCGAGACGTACGTACGGTGTGCCAGCMGCCGCGGTAA3′. The sequence for the reverse primer (R806) was 5′CAAGCAGAAGACGGCATACGAGATXXXXXXXXXXXXACGTACGTACCGGATACHVGGGTWTCTAAT3′. The reverse primer (R806) contained a 12-nucleotide error-correcting Golay code for sample identification. The XXXXXXXXXXXX represents a unique barcode sequence, the V represents either A, C, or G, M represents A or C, and W represents A or T^49^. Amplified products were concentrated and purified by solid-phase reversible immobilization and then quantified by electrophoresis using Agilent 2100 Bioanalyzer. Samples meeting the post-PCR minimum concentration were loaded into MiSeq Illumina for cluster formation and then sequenced for 250 cycles using primers designed for paired-end sequencing^9^. The paired-end reads were uploaded to the sequence read archive with the accession number SRP071608.

### Sequence analysis pipeline

A total of 41,123,342 paired-end reads were obtained and their sequencing quality assessed by FastQC^50^. The majority of the sequence processing was performed based on the Schloss MiSeq standard operating procedure using Mothur^51^. Briefly, paired sequences were merged into contigs followed by exclusion of sequences that had ambiguous base pairs, homopolymers longer than eight, or a read length greater than 275bp. Remaining sequences were trimmed using a 10bp sliding window with an average quality score of 28 and a minimal trimmed length of 200bp. Remaining high quality sequences were aligned to SILVA v119^52^. PCR errors were reduced using pre.cluster, and chimeras were detected and removed by UCHIME^53^. Subsequently, the high quality sequences were classified using Mothur’s implementation of the RDP classifier against the reference training set v14^54^. Sequences classified as Mitochondria, Chloroplasts, Archaea, Eukaryotes, or unclassified at the kingdom level were excluded. Remaining sequences were clustered at a distance of 0.03 (97% sequence similarity) using average neighbor clustering algorithm. After classifying the consensus taxonomy for each OTU, singletons were removed. To account for the presence of contaminating microbial 16S rDNA sequences in extraction kits and laboratory reagents, we removed all OTUs detected from control empty Eppendorf tubes. A final count of 15 NAF samples, along with 13 nipple skin, and 12 post-Betadine skin samples survived strict quality processing and subsequent background subtraction. The 15 NAF samples, nine from HC and six from BC, had a total of 31,968 reads distributed among 143 OTUs with a table density of 0.102. The 13 skin samples, eight from HC and five from BC, had a total of 31,679 reads distributed among 127 OTUs with a table density of 0.108. The post-Betadine skin samples, five from HC and seven from BC, had a total of 26,172 reads distributed among 119 OTUs with a table density of 0.114. Clinical information of the 12 patients whose NAF samples passed quality filtering and background subtraction is shown in Table 2. The table for the number of NAF samples attempted, collected, amplified, and passed quality filtering across clinical information are shown in Supplemental Table S6.

### Statistical analysis

To account for any bias caused by uneven sequencing depth, the least number of sequences present in any given sample from a sample category was selected randomly prior to calculating community-wide dissimilarity measures (rarefaction). 918 random sequences were chosen for the nipple skin analysis, comparing eight skin samples from HC vs. five from BC. 1164 random sequences were chosen for the analysis of the surgical scrubbed (Betadine) nipple skin microbiome, which compared five post-Betadine skin swabs from HC vs. seven from BC. 934 random sequences were chosen for NAF analysis, comparing nine NAF from HC vs. six from BC. Finally, 918 random sequences were chosen for the comparison between the nipple skin and the NAF samples, which had nine pairs collected from the same breasts: six from HC and three from BC. All Principal Coordinate Analyses (PCoA) were based on a Bray-Curtis dissimilarity using evenly sampled (rarefied) OTU abundances. To test for community compositional differences, we used analysis of variance (Adonis) from R’s vegan package^55^, which also noted that Adonis test is less sensitive to dispersion effects and is a more robust alternative to either analysis of similarities (ANOSIM) or multi-response permutation procedures (MRPP). To compare community-wide differences between paired nipple skin and NAF samples, the strata parameter was applied to restrict permutations within the patient variables and not across^55^. To test for differentially abundant OTUs between HC and BC samples, we employed a nonparametric Kruskal-Wallis test. As for comparing the OTU abundances between paired NAF and nipple skin, we instead used a paired Wilcoxon signed-rank test. The alpha-level cutoff was 0.05 and false discovery correction was not applied for comparing the OTU relative abundances. Observed OTU metric was used for alpha diversity calculations with ten permutations of random sampling at each sequencing depth. Taking the observed OTUs at the final sequencing depth, we performed a nonparametric t-test using Monte Carlo permutations to compare the diversity between samples from HC vs. BC. As for comparing the bacterial diversity between paired NAF and nipple skin samples, we instead used a paired two-tailed t-test. All statistical analyses were performed in R 3.2.3^56^.

### Functional Prediction with PICRUSt

Following the Mothur pipeline, the high quality filtered OTUs were aligned again, but this time against a closed reference Greengenes database (v13_8)^57^. The analysis is limited to bacteria whose genome, and therefore all of their genes and gene contents, are known. Due to the closed reference OTU picking, only 12 NAF samples, six from HC and six from BC, had sufficient 16S reads remaining. Next, we used PICRUSt package to computationally predict the gene contents from the 16S rRNA region, and subsequently, the pathways related to these genes^28^. Using PICRUSt, the picked OTUs were normalized to their copy numbers and their pathway abundances inferred from the Kyoto Encyclopedia of Genes and Genomes (KEGG)^58^. PICRUSt identified a total of 328 KEGG pathways and 6909 bacterial genes, of which we pre-selected 18 pathways to reduce false discovery rate from multiple hypothesis testing; the pathways were selected based on previous nutritional and microbiome studies in relation to colon cancer^20^,^21^,^22^,^23^,^24^,^25^,^26^,^27^. The selected 18 pathways were normalized to the sample with the lowest abundance, which was at a sampling depth of 26359. The predicted KEGG pathways between NAF from HC vs. BC were compared using Kruskal-Wallis test with Benjamini-Hochberg correction.

## Additional Information

**How to cite this article**: Chan, A. A. *et al*. Characterization of the microbiome of nipple aspirate fluid of breast cancer survivors. *Sci. Rep.* **6**, 28061; doi: 10.1038/srep28061 (2016).

## Supplementary Material

Supplementary Table

Supplementary Information

## Figures and Tables

**Table 1:** OTUs contributing to the KEGG ortholog Beta-Glucuronidase (K01195).

OTUs contributing to “Beta-Glucuronidase” (k01195)	Sum of relative abundances NAF from HC (n = 6)	Sum of relative abundances NAF from BC (n = 6)
p_Firmicutes; c_Clostridia; o_Clostridiales; f_Lachnospiraceae; g_Roseburia; unclassified	0	27
p_Bacteroidetes; c_Bacteroidia; o_Bacteroidales; f_Rikenellaceae; unclassified; unclassified	0	265
p_Acidobacteria; c_Chloracidobacteria; o_RB41; f_Ellin6075; unclassified; unclassified	74	231
p_Bacteroidetes; c_Bacteroidia; o_Bacteroidales; f_Bacteroidaceae; g_Bacteroides; s_uniformis	0	1
p_Firmicutes; c_Bacilli; o_Bacillales; f_Paenibacillaceae; g_Paenibacillus; s_amylolyticus	0	3
p_FBP; unclassified; unclassified; unclassified; unclassified; unclassified	0	226
p_Actinobacteria; c_Thermoleophilia; o_Solirubrobacterales; unclassified; unclassified; unclassified	13	0
p_Bacteroidetes; c_Bacteroidia; o_Bacteroidales; f_Bacteroidaceae; g_Bacteroides; s_caccae	13	0
Total Predicted Beta-Glucuronidase	99	753

List of the eight OTUs (classified to Greengenes v13_8) whose PICRUSt prediction corresponds to the KEGG ortholog Beta-Glucuronidase (k01195). The “sum of relative abundances” indicates how much of the “Total Beta-Glucuronidase” is predicted by that OTU.

**Table 2:** Clinical information for women whose NAF samples passed quality filtering.

		Healthy Control Women (HC)	Women with a History of Breast Cancer (BC)
Age	average (range)	52 (41 to 64)	58 (52 to 66)
Caucasian	n (%)	6 (100%)	6 (100%)
BMI	average ± SD (range)	25.6 ± 8.1 (19.5 to 35.1)	24.3 ± 4.6 (20.6 to 31.3)
Menopausal status	n (%)		
Pre-menopausal		2 (33.3%)	1 (16.7%)
Peri-menopausal		1 (16.7%)	1 (16.7%)
Post-menopausal		3 (50.0%)	4 (66.6%)
Estrogen Receptor (ER)	n (%)		
Positive		NA	3 (50.0%)
Negative		NA	3 (50.0%)
Progesterone Receptor (PR)	n (%)		
Positive		NA	1 (16.7%)
Negative		NA	2 (33.3%)
Unknown		NA	3 (50.0%)
Human Epidermal Growth Factor Receptor 2 (HER2)	n (%)		
Positive		NA	3 (50.0%)
Negative		NA	3 (50.0%)
Breast Feed	n (%)		
Yes		2 (33.3%)	3 (50.0%)
No		4 (66.7%)	2 (33.3%)
Unknown		0 (00.0%)	1 (16.7%)
Antibiotics in the Past Year	n (%)		
Yes		3 (50.0%)	1 (16.6%)
No		3 (50.0%)	4 (66.7%)
Unknown		0 (00.0%)	1 (16.7%)
Hormone therapy/Oral contraceptive	n (%)		
Yes		3 (50.0%)	2 (33.3%)
No		3 (50.0%)	3 (50.0%)
Unknown		0 (00.0%)	1 (16.7%)
Recent Breast Exposure to Mouth by Breast-feeding/Sex	n (%)		
Yes		3 (50.0%)	3 (50.0%)
No		3 (50.0%)	2 (33.3%)
Unknown		0 (00.0%)	1 (16.7%)
Mother had Breast Cancer	n (%)		
Yes		2 (33.3%)	1 (16.7%)
No		4 (66.7%)	4 (66.7%)
Unknown		0 (00.0%)	1 (16.7%)
Dense Breast Tissue on Mammogram	n (%)		
Yes		4 (66.7%)	3 (50.0%)
No		2 (33.3%)	1 (16.7%)
Unknown		0 (00.0%)	2 (33.3%)

Demographics for 6 healthy control women and 6 women with a history of breast cancer whose NAF samples had sufficient reads remaining after quality filtering and background subtraction removing all OTUs detected in the empty control Eppendorf tube.
