# Supplementary material for: Characterization of the microbiome of nipple aspirate fluid of breast cancer survivors
Source: Sci Rep. 2016 Jun 21;6:28061. doi: 10.1038/srep28061 (PMC4914981; doi:10.1038/srep28061)
Supplement: Supplementary Information [file srep28061-s2.doc]

**Characterization of the microbiome of nipple aspirate fluid of breast cancer survivors**

Alfred A. Chan^1^**^*^**(ChanA@JWCI.org), Mina Bashir^2,3^**^*^** (mina.bashir@medunigraz.at), Magali N. Rivas^1^**^*^** (Magali.NovalRivas@cshs.org), Karen Duval^4,5^ (KDuvall@mednet.ucla.edu), Peter A. Sieling^1^ (Peter.Sieling@NantBio.com), Thomas R. Pieber^3^ (thomas.pieber@medunigraz.at), Parag A. Vaishampayan^2^ (vaishamp@jpl.nasa.gov), Susan Love^5^ (slove@drsusanloveresearch.org), and Delphine J. Lee (LeeDJ@JWCI.org)^1^

^1^ Dirks/Dougherty Laboratory for Cancer Research, Department of Translational Immunology, John Wayne Cancer Institute at Providence Saint John’s Health Center, Santa Monica, CA, USA. ^2^ Biotechnology and Planetary Protection Group, Jet Propulsion Laboratory, California Institute of Technology, Pasadena, CA, USA. ^3^ Division of Endocrinology and Metabolism, Medical University of Graz, Graz, Austria. ^4^ Breast Center at the University of California Los Angeles (UCLA), Westwood, Los Angeles, CA, USA. ^5^ Doctor Susan Love Research Foundation, Encino, CA, USA.

***** These first authors contributed equally to this work.

**Corresponding Author:** Delphine J. Lee – Dirks/Dougherty Laboratory for Cancer Research, Department of Translational Immunology, John Wayne Cancer Institute at Providence Saint John’s Health Center. 2200 Santa Monica Boulevard, 90404 Santa Monica, CA, USA

**Email:** [LeeDJ@JWCI.org](mailto:LeeDJ@JWCI.org)

**PROCEDURE**

1. Consent subject
2. Subject changes into exam gown
3. Heat up purple breast warmer 3 minutes (2x 1.5 min each) and fill metal tray with ice
4. Subject fills out questionnaire while wearing breast warmer outside of gown
5. Measure subject height and weight
6. Escort subject into procedure room and subject puts on booties and mask
7. Get subject situated on table and make sure she is comfortable
8. Ask subject to massage breasts
9. Clinician puts on hat, mask, booties
10. Assistants put on isolation gown, hat, mask, gloves, and booties
11. Open sterile glove package for clinician; clinician puts on sterile gloves
12. Open swab package for clinician
13. For left baseline skin sample, swab nipple and areola; place in collection tube held by lab personnel; place tube on ice
14. Open sterile glove package for clinician; clinician puts on new pair of sterile gloves
15. Open swab package for clinician
16. For right baseline skin sample, swab nipple and areola; place in collection tube held by lab personnel; place tube on ice
17. On the sterile side of the glove package, drop gauze and add Nuprep
18. Using the same pair of sterile gloves, scrub left breast with Nuprep to remove keratin plugs and scrub right breast with Nuprep to remove keratin plugs
19. Give clinician scrub brush
20. Clinician performs sterile surgical hand scrub
21. While clinician scrubs in, cover subject with gown and ask subject to massage breasts
22. Open sterile gown package for clinician; clinician places on metal tray
23. Clinician dries hands using sterile towel and puts on sterile gown with the help of assistant
24. Open sterile glove package for clinician; clinician puts on gloves
25. Open sterile large nonfenestrated drape and place over the subject’s abdomen and legs
26. Open Povidone-Iodine swab package in sterile fashion for clinician
27. Sterilize left nipple taking a wide swath of the surrounding area
28. Repeat using a new Povidone-Iodine swab package
29. Open Povidone-Iodine swab package in sterile fashion for clinician
30. Sterilize right nipple taking a wide swath of the surrounding area
31. Repeat using a new Povidone-Iodine swab package
32. Maintaining sterility, tear open swab package for clinician
33. Open swab package for clinician
34. For the left post-sterilization betadine sample, swab left nipple and areola; place in collection tube held by lab personnel; place tube on ice
35. Open swab package for clinician
36. For the right post-sterilization betadine sample, swab right nipple and areola; place in collection tube held by lab personnel; place tube on ice
37. Open sterile glove package for clinician; clinician puts on gloves
38. Open fenestrated drape package for clinician
39. Clinician puts fenestrated drape over subject’s breast
40. Put sterile drape on tray
41. Drop aspirator and syringe onto tray
42. Maintaining sterility, obtain NAF
43. Record location, color and size of NAF in subject chart; take picture if desired
44. Open swab package for clinician
45. For NAF sample, swab nipple; place in collection tube held by lab personnel; place tube on ice
46. Repeat NAF collection process (steps 43-46) 2x more for a total of 3 aspirations
47. Throw out drape, aspirator and syringe
48. Open sterile glove package for clinician; clinician puts on gloves
49. Repeat NAF collection process (steps 37-48) on other breast

**healthy subjects sample both breasts; breast cancer subjects sample contralateral only
